# 686 A Balanced Approach to Scar Management of the Complex Infant Facial Burn

**DOI:** 10.1093/jbcr/iraf019.315

**Published:** 2025-04-01

**Authors:** Debbie Minter, Stacey Foliente, Ingrid Parry, Tina Palmieri, Soman Sen, Kathleen Romanowski, Jason Heard

**Affiliations:** Shriners Children’s - Northern California; Shriners Children’s - Northern California; Shriners Children’s - Northern California; University of California, Davis and Shriners Hospitals for Children; University of California, Davis and Shriners Hospitals for Children; University of California, Davis and Shriners Hospitals for Children; University of California, Davis

## Abstract

**Introduction:**

Managing the scarring of infant facial burns poses a significant challenge to burn clinicians. Scar management in this age group is unique due to rapid sensorimotor development and craniomaxillofacial growth. From 6-12 months, the infants’ brain volume doubles and head circumference increases by 1 cm per month. Best evidence practice for scar management includes the use of compression 23 hours/day yet the literature guiding its use in this special population is sparse. In this report, we demonstrate that scar outcomes in this age group can be optimized while allowing for cranial growth with a balanced treatment approach.

**Methods:**

This is a complex case involving a 6-month-old male with 42% TBSA, including extensive facial burns and skin grafting. His facial scar management program included the use of soft cloth surgical tape, silicone, intraoral stretching and massage. A fabric compression face mask was worn most nights for 6-8 hours and a transparent face mask with a beanie strap system was used on average 2 hours/day. Cranial growth was monitored utilizing serial head circumference, cranial vault asymmetry index (CVAI), and cephalic index (CI) measurements. Functional status was assessed with the Mouth Impairment and Disability Assessment (MIDA) and facial scars were rated with the Patient Observer Scar Assessment Scale (POSAS).

**Results:**

At follow-up at age 22 months, the patient scored 13/64 on the functional activities portion of the MIDA showing that he was able to do all oral activities with slight to no difficulty. He scored 4/16 on the satisfaction/social impact portion indicating that his father was “very satisfied” with the facial aesthetic and functional outcomes. On the POSAS, the father rated the facial scars overall as 9/10 leaning towards “very different” while the therapist’s overall opinion of the facial scars was 3/10 leaning away from “worst scar imaginable”. Serial head circumference measurements indicated that this patient was following his cranial growth curve on the 50th percentile. CVAI of 0.6 % indicates a symmetrical head while CI of 76% represents a normocephalic shaped skull.

**Conclusions:**

The treatment of infant facial burns requires awareness that standard therapeutic interventions for hypertrophic scarring could have adverse consequences on craniomaxillofacial growth and potentially affect brain volume and development. To our knowledge, this is the first case study to demonstrate that the use of pressure therapy in this age group is possible when it is balanced with the unique needs of this cohort and utilizes standardized measures to ensure adequate cranial growth.

**Applicability of Research to Practice:**

More research is needed on the effects of pressure therapy on craniomaxillofacial growth and the brain development of infants. Patients in this age group would benefit from the development of evidence-based recommendations for the use of pressure therapy.

**Funding for the Study:**

N/A

